# Effects of proprioceptive exercises on pain and function in chronic neck- and low back pain rehabilitation: a systematic literature review

**DOI:** 10.1186/1471-2474-15-382

**Published:** 2014-11-19

**Authors:** Michael A McCaskey, Corina Schuster-Amft, Brigitte Wirth, Zorica Suica, Eling D de Bruin

**Affiliations:** Research Department, Reha Rheinfelden, Salinenstrasse 98, 4310 Rheinfelden, Switzerland; Department of Health Sciences and Technology, ETH Zurich, Wolfgang-Pauli Str. 27, 8093 Zurich, Switzerland; Department of Epidemiology, CAPHRI School for Public Health and Primary Care, Maastricht University, PO Box 616, 6200 Maastricht, MD The Netherlands; Centre for Evidence Based Physiotherapy, Maastricht University, PO Box 616, 6200 Maastricht, MD The Netherlands; Institute of Rehabilitation and performance Technology, Bern University of Applied Sciences, Pestalozzistrasse 20, 3400 Burgdorf, Switzerland

**Keywords:** Proprioception, Low back pain, Neck pain, Proprioceptive training, Systematic review

## Abstract

**Background:**

Proprioceptive training (PrT) is popularly applied as preventive or rehabilitative exercise method in various sports and rehabilitation settings. Its effect on pain and function is only poorly evaluated. The aim of this systematic review was to summarise and analyse the existing data on the effects of PrT on pain alleviation and functional restoration in patients with chronic (≥3 months) neck- or back pain.

**Methods:**

Relevant electronic databases were searched from their respective inception to February 2014. Randomised controlled trials comparing PrT with conventional therapies or inactive controls in patients with neck- or low back pain were included. Two review authors independently screened articles and assessed risk of bias (RoB). Data extraction was performed by the first author and crosschecked by a second author. Quality of findings was assessed and rated according to GRADE guidelines. Pain and functional status outcomes were extracted and synthesised qualitatively and quantitatively.

**Results:**

In total, 18 studies involving 1380 subjects described interventions related to PrT (years 1994–2013). 6 studies focussed on neck-, 12 on low back pain. Three main directions of PrT were identified: Discriminatory perceptive exercises with somatosensory stimuli to the back (pPrT, n = 2), multimodal exercises on labile surfaces (mPrT, n = 13), or joint repositioning exercise with head-eye coordination (rPrT, n = 3). Comparators entailed usual care, home based training, educational therapy, strengthening, stretching and endurance training, or inactive controls. Quality of studies was low and RoB was deemed moderate to high with a high prevalence of unclear sequence generation and group allocation (>60%). Low quality evidence suggests PrT may be more effective than not intervening at all. Low quality evidence suggests that PrT is no more effective than conventional physiotherapy. Low quality evidence suggests PrT is inferior to educational and behavioural approaches.

**Conclusions:**

There are few relevant good quality studies on proprioceptive exercises. A descriptive summary of the evidence suggests that there is no consistent benefit in adding PrT to neck- and low back pain rehabilitation and functional restoration.

**Electronic supplementary material:**

The online version of this article (doi:10.1186/1471-2474-15-382) contains supplementary material, which is available to authorized users.

## Background

Treatment of chronic pain has always been, and still is, a challenging field for therapists and researchers alike. Treatment is particularly problematic in patients who report significant pain with associated limitations for daily activities, but present with no structural or organic causes. More than 80% of all chronic low back pain (LBP) patients referred to physiotherapy are diagnosed with such non-specific LBP (NSLBP) causing corresponding figures in medical costs [1]. Despite the progress in the understanding of pain and its management, NSLBP is still stated as the leading cause for years lived with disability, worldwide [2]. With the expected increase of this global burden over the next decades [3] there is still an urgent need for effective NSLBP treatment.

According to a recent, integrative model of chronic NSLBP development, changes in the amount and pattern of movements is at the beginning of pain chronification processes [4]. Flawed movements caused by either fear in response to an acute pain episode or environmental conditions (e.g. repetitive movements at work, or sustained postural misalignment) are believed to lead to impaired sensorimotor control and have been suggested to contribute to tissue pathology in NSLBP [4–10].

The relationship of pain and changes in motor control has been shown in several studies [11–17] and is seen as a protective reaction of the body to limit provocation of the painful area [9]. This, in the long run, can cause further damage, exacerbate the symptoms through peripheral and central nervous system sensitization (lowering of pain threshold), and promote dysfunctional movement patterns [4, 10, 18]. A commonly described theory suggests that reduced afferent variability from peripheral proprioceptive receptors may cause neuromuscular deficiencies. If not restored, this constant malfunctioning of neuromuscular control and flawed regulation of dynamic movements may lead to inappropriate muscular activity (i.e. over- or under-utilization) [19–22]. This is thought to contribute to taut muscles, imbalanced muscle activation, poor posture, and ultimately to musculoskeletal pain in lumbar regions [4, 10, 19–21, 23]. Psychosocial factors can contribute to decreased physical activity and enforce the “vicious cycle” described above [4].

This ‘functional pathology’ theory [10] is supported by several findings in current literature. It has been shown that patients with NSLBP have modified muscle recruitment patterns [4, 24–26], reduced postural robustness [6], inappropriate variability in postural control [27–30] and seem to rely more on distal proprioception [6] due to impaired proprioception from proximal segments [6, 31]. Such deficits in the motor system occur early in the history of onset of pain [32] and have been associated with a decreased ability of the central nervous system to process proprioceptive inputs [33].

Proprioception is defined as afferent information that contributes to conscious muscle sense, total posture, and segmental posture [34]. Proprioceptive feedback influences movement accuracy, timing of the onset of motor commands, and adapting to movement situations that require the use of non-preferred coordination patterns [35]. Maintaining proprioceptive integration in neuromuscular control of posture has been identified as important resource for unimpaired and pain-free participation of daily activities [36]. Furthermore, improvement of neuromuscular function of the trunk has been suggested to be more important than strengthening in patients with LBP [15, 26, 37] Consequently, neuromuscular rehabilitation techniques addressing sensory deficiencies through increased proprioceptive challenge have emerged in recent years and have received increasing therapeutic attention [22, 23, 38].

Restitution of healthy neuromuscular motor patterns and increased sensory input variation is thought to reduce mechanical stress through improved muscular coordination and may prevent recurrence of NSLBP [32, 39]. So far only poorly evaluated, potential benefits are expected from proprioceptive exercises and joint position training to reduce pain and disability [40]. These exercises would generally entail balance training and the use of labile platforms to repeatedly provoke sensory receptors and subsequent integration of these perceptions in the spinal cord, pons, and higher cortical areas [41, 42]. This is thought to lead to increased perception of joint position- and motion, hence supporting unconscious joint stabilization through reflex which again maintains healthy posture and balance [23].

There is an increasing amount of used expressions and a wide variability in the nature, mode and context of methods attempting similar effects. Moreover, there has been some doubt on whether PrT can improve proprioceptive acuity in a functional way at all. In a recent narrative review, Ashton-Miller et al. outlined a row of concerns (e.g. lack of neurophysiological evidence) about the validity of current proprioceptive exercises [43]. Although many therapists and clinicians report successful treatment cases, the exact effect and validity of sensorimotor interventions is still discussed controversially [43, 44]. Accordingly, European Guidelines on the management of chronic nonspecific LBP do not include recommendations for PrT [45].

However, maintaining variability of the collective sensory input is the basis of the dynamics behind human movement, allowing adjustable functional behaviour [46]. Although it remains unclear whether reduced proprioception is the cause [5] or the result of musculoskeletal pain [47, 48], improvement of pain has been linked to changes in neural activation [49] and psychological changes [50].

This article systematically reviews sensorimotor training procedures that target maladaptive changes in patients suffering from chronic non-specific neck- or low back pain. The main objective is to investigate current evidence supporting the effectiveness of integrated sensorimotor training concepts with proprioceptive elements in musculoskeletal pain rehabilitation that aim at reducing pain and improving functional status. Furthermore, studies reporting positive outcomes (improvement of functional status and reduced pain) shall be identified to describe what practical features of sensorimotor training are necessary to be successful and effective.

## Methods

Only randomised controlled trials were included for this systematic review (SR). Titles retrieved from electronic search, were screened by two authors (MM and CS). To qualify as an eligible study, participants had to be of adult age (>18 years), present with chronic non-specific musculoskeletal neck- or low back pain (at least three months), including whiplash-associated disorders. Only studies declaring clinical examination or interview assessment of pain were included. Exclusion criteria were neurological deficits related to peripheral or central nerve damage, vestibular diseases, osteoarticular diseases (e.g. rheumatoid arthritis), fractures, and tumours. No restrictions regarding gender, ethnicity, language, or clinical setting (in-patients or out-patients) were made. Pain during or after pregnancy, complex regional pain syndrome, headache alone, and fibromyalgia were also added to the exclusion criteria.

The effectiveness of PrT was compared to other forms of exercise, educational interventions, and to inactive control groups. All variations of PrT, where active participation of the patient was described (balancing- and perturbation exercises, joint repositioning) were included. Passive methods, where patients did not actively have to respond to peripheral feedback (e.g. exercises on vibrating platforms), were excluded. Also Yoga, Pilates, and Global Postural Re-education (GPR) were not included. The search was not limited to one kind of comparator. All forms of control-interventions were included (e.g. massage or educational, strengthening exercises, endurance training, etc.). The a-priori defined research question and protocol is provided as Additional file 1. An overview of the eligibility criteria of included studies can be found in the Additional file 2.

### Information sources and search strategy

The Cochrane Central Register of Controlled Trials CENTRAL (The Cochrane Library 2014, Issue 2) and further databases were searched from their respective inception to February 2014 (MEDLINE via Ovid, EMBASE, CINAHL via EBSCOhost, SportDISCUS, and SCOPUS). Medline and SCOPUS were combined in order to cover the gaps in citations published prior to 1996. Reference lists of included articles were reviewed for further citations. A combination of medical subject headings (MeSH: Musculoskeletal Pain, Low Back Pain, Fibromyalgia, Reflex Sympathetic Dystrophy, Joint Instability, Shoulder Pain, Myofascial Pain Syndromes) and search terms (pain, discomfort, trouble, hurt, muscle imbalance, muscle stiffness, shoulder-, neck-, pelvic- or back pain) was used for the population. For the intervention, the following search terms were combined: sensory motor or sensorimotor, proprioceptive, balance, postural, coordination, motor control, cybernetic, stabilising. The search was not restricted to specific outcomes. The first search was executed on December 6^th^ 2012 by a Life Science librarian from a medical library and, as an update of the search, repeated with saved searches on February 25^th^ 2014. An example search is provided as Additional file 3.

### Study selection

De-duplication had been performed by the assigned librarian when two review authors (MM & CS) independently screened articles for inclusion criteria according to standardised protocol. Titles, abstracts, and full texts were screened sequentially. Disagreement of selected full texts was resolved with mutual consent. If authors could not agree upon the issue, the last author (EdB) was consulted to decide on in- or exclusion.

Foreign language full texts were not excluded immediately. Instead, the authors or institutions were contacted to elucidate whether a translated version of the article was available. With no English or German version available, the reference was excluded.

### Data collection process

One review author (MM) extracted all data and recorded it on a standardised data-extraction form based on the template by the Cochrane Pain, Palliative & Supportive Review Group [51]. The data extraction form was pilot- tested on four studies, and refined accordingly. A second review author (CS) crosschecked the extracted data on three randomly selected studies (randomised with random number generator on Microsoft Excel). Disagreements were resolved by discussion between the two review authors; if no agreement could be reached, it was planned a third author (EdB) would decide. Inter-rater agreement above 90% was deemed satisfactory. The extracted data included study design and methodology (including randomisation procedures and settings), participants’ characteristics, details of the interventions, dropouts and withdrawals, and outcome measure's change from baseline to endpoint. In case of inconclusive data (e.g. only graphical presentation, missing variance of change), the original authors or institutions were contacted to obtain missing details.

### Risk of bias in individual studies

**Figure 1:**
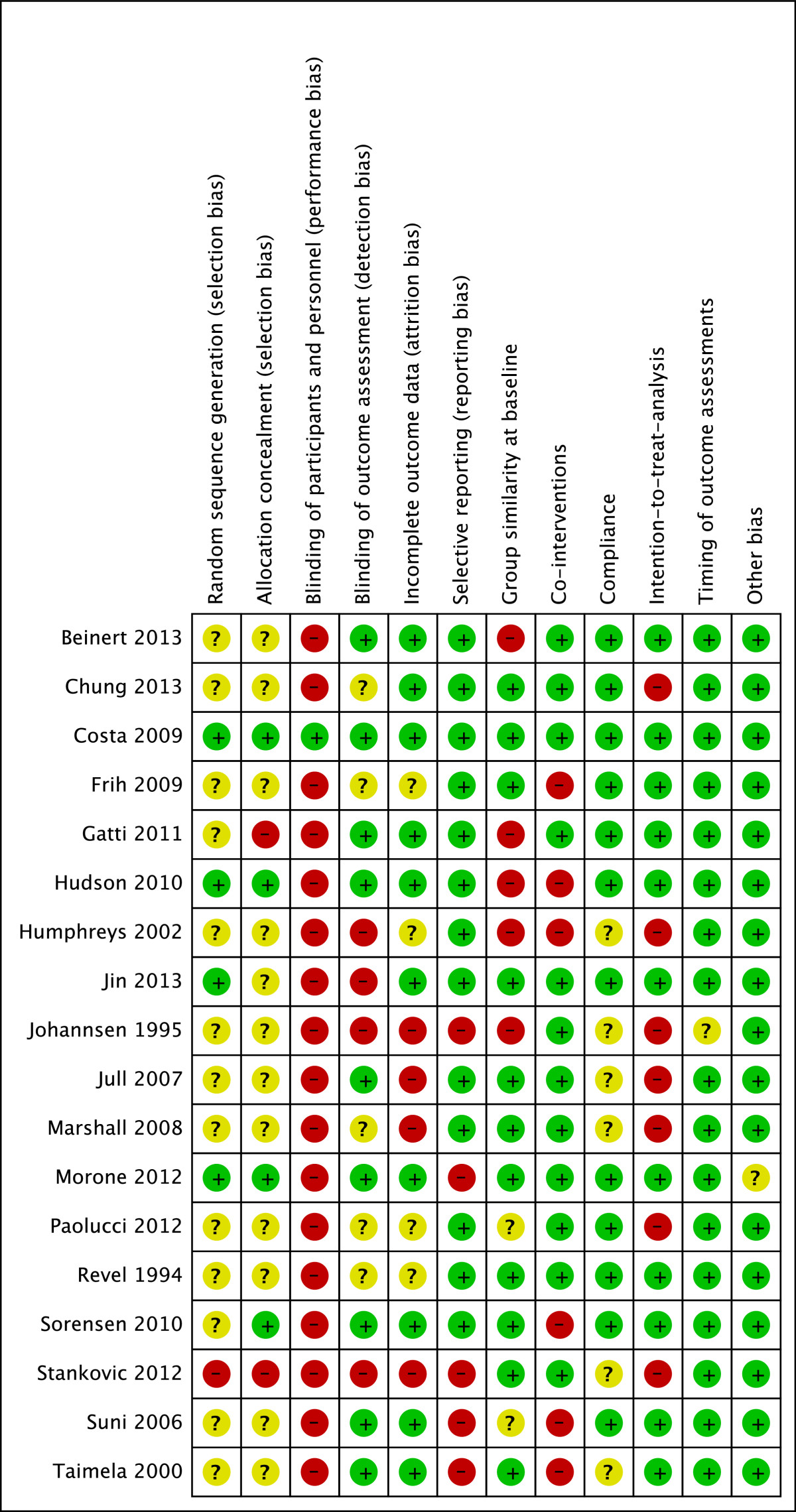
**Risk of bias summary: review authors’ judgements about each risk of bias item for each included study.** (+) = Low risk of bias; (−) = high risk of bias; (?) = unclear risk of bias.

As the Cochrane Collaboration discourages the use of summary scores for RoB assessment, two reviewers (MM & CS) independently applied the Cochrane Collaborations tool to judge the risk of over- or underestimating the effects of an intervention [52]. In total, twelve domains of bias were rated for every study, each domain having three rating categories (Figure 1): (1) low RoB, (2), high RoB and (3) unclear RoB. Rating (1) is unlikely to alter the results significantly, (2) seriously weakens confidence in the results, and (3) raises some doubt about the results. With insufficient information on an item the score given was ''unclear''. As suggested by the Cochrane Back Review Group (CBRG) [53], more topic-specific sources of biases were assessed. Specifically, baseline similarity, equal dose and frequency of co-interventions, compliance, adherence to intention-to-treat analysis, and timing of outcome assessment were compared between the groups. The arbitration of a third reviewer (EdB) was used in the event of any disagreement between the reviewers (MM & CS) for both ratings. Percentage agreement and Cohen's kappa were calculated and interpreted in accordance with Landis and Koch's benchmarks for assessing the agreement between raters: poor (0), slight (0.0 to 0.20), fair (0.21 to 0.40), moderate (0.41 to 0.60), substantial (0.61 to 0.80), and almost perfect (0.81 to 1.0) [54].

### Analysis and GRADE approach

The review topic includes a wide range of intervention methods (different concepts of sensorimotor training) and participants (non-specific musculoskeletal back- or neck- pain). The collected data is therefore prone to high heterogeneity, which discourages a meta-analysis. To test for statistical heterogeneity, data was entered into Review Manager (RevMan5, The Cochrane Collaboration, Oxford, UK) and Microsoft Excel (2010) to calculate mean differences (MD), standard deviation (SD), confidence intervals (CI), and p-values (p). Missing SDs and MDs were calculated according to the Cochrane Handbook for Systematic Reviews [55], if applicable.

Funnel plots of the trial's SMD were evaluated using Review Manager (RevMan5, The Cochrane Collaboration, Oxford, UK). Asymmetry in a funnel plot indicates possible non-publication of small trials with negative results [55].

Interventions were compared based on clinical homogeneity (study population, types of treatment, outcomes and measurement instruments) and choice of proprioceptive training modality. Trials that used the same tools for outcome assessment were compared using the mean difference (MD) to allow direct comparison of the results. If trials within the same comparison used different measurement tools for the same outcome, the standardized mean difference (SMD) was calculated using random-effect models. If only graphs were available, the mean scores and standard deviations (SD) had to be estimated from the illustrations. If missing SDs of change were not available, SDs of post-treatment scores were used [55]. If SDs for outcomes were not reported at all, they were estimated using the mean SD weighted by the relevant treatment group’s sample size across all other trials that reported SDs for same outcome [53].

The GRADE (Grades of Recommendation, Assessment, Development and Evaluation) approach was used to rate the overall quality of the evidence and the strength of the recommendations [53]. Following the CBRG method guidelines [53], five domains of quality were rated for each comparison: (1) Limitations of study design (>25% of participants from studies with high risk of bias); (2) Inconsistency (i.e. opposite direction of effects and/or significant statistical heterogeneity); (3) Indirectness (e.g. only one gender or specific age group included); (4) Imprecision (e.g. too few participants or only one study included); (5) Publication bias across all trials. Rating was conducted by one author (MM) and crosschecked by a second (ZS) on randomly selected comparisons.

The four-point rating scale ranged from ‘High quality’ on one end to ‘Very low quality’ on the other end. To qualify as high quality evidence, more than 75% of the RCTs within a comparison had to be judged to have no limitations of study design, have consistent findings among multiple studies, present direct (generalizable) and precise data, without known or suspected publication bias. The quality of the summary of findings was rated as moderate if one, low if two, and very low if three of the criteria were not met. The definitions of quality of the evidence were adopted from Guyatt et al. [56]:

*High quality:* Further research is very unlikely to change our confidence in the estimate of effect. *Moderate quality:* Further research is likely to have an important impact on our confidence in the estimate of effect and may change the estimate. *Low quality:* Further research is very likely to have an important impact on our confidence in the estimate of effect and is likely to change the estimate. *Very low quality:* We are very uncertain about the estimate.

## Results

### Study selection

**Figure 2:**
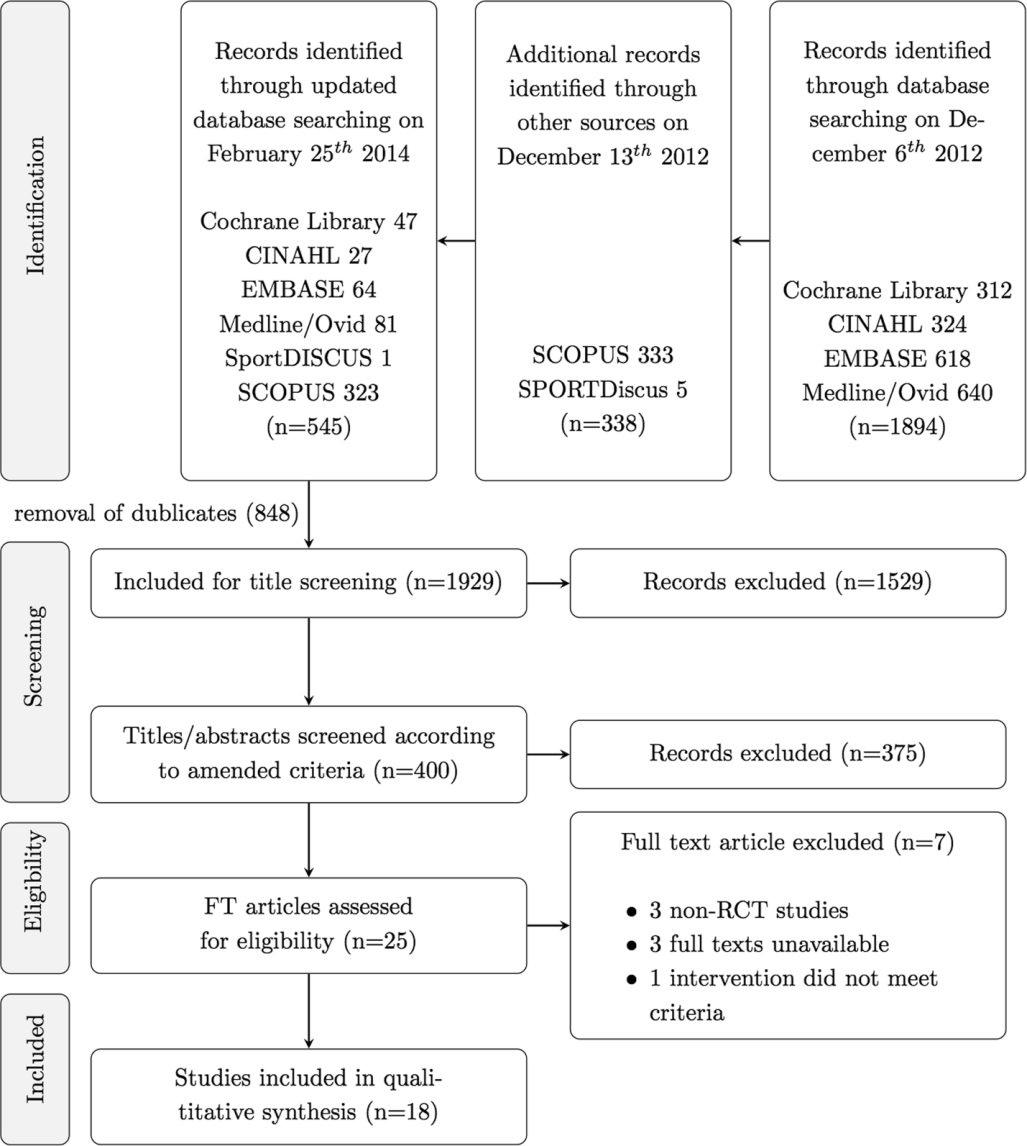
**Screening progress flow chart.** n = number of references; RCT = randomized controlled trials, FT = full-texts.

After adjusting for duplicates, the latest search of the databases provided a total of 1929 citations. Of these, 1901 were discarded after reviewing titles and abstracts, clearly showing that these papers did not meet the criteria. Three additional studies were discarded because full texts of the study were not available or the papers could not be feasibly translated into English. The full texts of the remaining 25 citations were examined in more detail. Finally, 18 studies met the inclusion criteria and were included in the SR. No unpublished relevant studies were obtained (Figure 2).

### Study characteristics

The included 18 studies, all describing interventions related to PrT, were published between 1994 and 2013, all of them in English. The reports describe randomised controlled trials with one to three comparators (Table 1).

**Table 1:** **Overview of included studies and descriptive study data**

Reference	Participants		Intervention	Comparator	Outcome	Group effect
Beinert [71]	Total N	34	5 weeks, 15×15 min., three balance exercises with increasing difficulty: single leg, tandem, and standing on a wobble board	No intervention; participants were instructed to maintain physical activity as usual	Numeric Pain Rating Scale (NRS)	↗
CH, 2013	Age:	23			Head relocation from neutral position	↗
	Pain area:	NP			Pre-rotated head relocation	↗
	Cl. confirmed:	No				
	Gender (f/m):	nA				
Chung [73]	Total N	24	8 weeks, 3 sessions/week (duration not specified), 10 Min. warm-up followed by four lumbar stabilisation exercises on a small gymnastics ball	8 weeks, 3 sessions/week (duration not specified), 10 Min. warm-up followed by four lumbar stabilisation exercises on a mat	Pain intensity VAS	=
KR, 2013	Age:	38			Oswestry Disability Index (ODI) Weight bearing (postural sway) Multifidus cross section L2 and L3 Multifidus cross section L4 and L5	↗
	Pain area:	LBP				=
	Cl. confirmed:	Yes				=
	Gender (f/m):	11/13				↗
Costa [69]	Total N	154	8 weeks, 12×30 min. motor control exercise to improve function of specific muscles of the low back and control of posture and movement	8 weeks, 12×25 min. Shortwave Diathermy, Ultrasound (placebo)	Numeric Pain Rating Scale (NRS)	=
AU, 2011	Age:	54			Patient Specific Functional Scale (PSFS)	↗
	Pain area:	LBP			Global Impression of Recovery (GPE)	↗
	Cl. confirmed:	No			Roland Morris Score (RMS)	↗
	Gender (f/m):	28/51				
Frih [67]	Total N	107	4 weeks, 28×30 min. home-based rehabilitation programme: postural control, stretching and strengthening exercises	4 weeks, 12×90 min. standard rehabilitation programme: analgesic, electrotherapy, pain management, stretching, proprioceptive, and strengthening exercises	Pain intensity VAS	↗
TN, 2004	Age:	36			MacRae Schöber Index	=
	Pain area:	LBP			Finger-to-Floor (FTF) distance	=
	Cl. confirmed:	Yes			Thigh-leg (TL) angle	=
	Gender (f/m):	80/25			Shirado Test	↗
					Sorensen Test	=
					Quebec Functional Index	=
Gatti [63]	Total N	179	5 weeks, 10×60min. treadmill (15 min.), flexibility (30 min.), and trunk balance (15 min.) exercises	5 weeks, 10×60 min. treadmill (15 min.), flexibility (15 min.), and strengthening (15 min.) exercises	Pain Intensity VAS (0 to 100)	=
IT, 2009	Age:	58			Roland Morris Score (RMS)	↗
	Pain area:	LBP			Quality of Life, physical (SF-12p)	↗
	Cl. confirmed:	Yes			Quality of Life, mental (SF-12m)	=
	Gender (f/m):	11/23				
Hudson [62]	Total N	14	6 weeks, 6×60 min. multimodal treatments: coordinative, proprioceptive, strengthening, and educational components	6 weeks, 6-8×20 min. usual care (any combination of exercise, education, mobilisations, manipulations, electrotherapy, or acupuncture)	Neck Disability Index (NDI)	=
UK, 2010	Age:	43			Numerical Pain Rating Scale (NRS)	=
	Pain area:	NP				
	Cl. confirmed:	No				
	Gender (f/m):	8/4				
Humphreys [57]	Total N	63	4 weeks, 56 treatments (twice a day, duration not specified) coordinative exercises (eye-head-neck coordination)	No intervention	Head Repositioning HRA	↗
UK, 2002	Age:	nA			Self-reported Pain Intensity (VAS)	↗
	Pain area:	NP				
	Cl. confirmed:	No				
	Gender (f/m):	nA				
Jin [72]	Total N	14	4 weeks, 20×40 Min. Six different quadruped exercises on a wobble board	4 weeks, 20×40 Min. physical therapy (20 Min. hot press; 5 Min. ultrasound; 15 Min. transcutaneous electrical nerve stimulation)	Pain intensity VAS	↗
KR, 2013	Age:	45			Oswestry Disability Index (ODI)	↗
	Pain area:	LBP			Anticipatory postural adjustment	↗
	Cl. confirmed:	No				
	Gender (f/m):	8/6				
Johannsen [64]	Total N	40	12 weeks, 24×60min. warm up (10 min.) coordinative, proprioceptive, balance, and stability exercises (40 min.), stretching (10 min.)	12 weeks, 24×60min. endurance (10 min.), dynamic strengthening exercises (40 min.), and stretching (10 min.)	Isokinetic back strength (KinCom II)	=
DK, 1999	Age:	38			Patient's general assessment	=
	Pain area:	LBP			Pain score (0-8)	=
	Cl. confirmed:	Yes			Mobility score (cm)	=
	Gender (f/m):	93/120				
Jull [58]	Total N	64	6 weeks, 84×10 min. (twice per day) proprioceptive training (head relocation practice), coordinative exercises (eye/head coordination)	6 weeks, 84×10 min. (twice per week) strengthening of deep cervical flexor muscles	Joint Position Error (JPE)	=
AU, 2005	Age:	41			Left JPE	↙
	Pain area:	NP			Right JPE	=
	Cl. confirmed:	Yes			Extension Neck Disability Index (NDI)	=
	Gender (f/m):	64/0			Numerical Rating Scale (NRS)	=
Marshall [70]	Total N	54	4 weeks usual care, then 12 weeks, 12 proprioceptive and strengthening exercises using the therapy ball (Swiss ball)	4 weeks usual care, then 12 weeks home based therapy regime based on commonly recommended low back strengthening exercises	Oswestry Disability Index	=
NZ, 2008	Age:	35			FR-Response	=
	Pain area:	LBP			Feed-forward activation assessment	=
	Cl. confirmed:	No				
	Gender (f/m):	27/27				
Morone [60]	Total N	75	4 weeks, 12×45 min. perceptive rehabilitation with proprioceptive components	3 weeks, 10 sessions (duration per session not reported), Back School based on re-education of breathing, stretching, postural, and strengthening exercises	Visual Analogue Scale	↗
IT, 2011	Age:	55			McGill Pain Rating Index	↗
	Pain area:	LBP			Oswestry Disability Index	=
	Cl. confirmed:	Yes			Wadell Disability Index	=
	Gender (f/m):	54/21				
Morone [60]	Total N	75	4 weeks, 12×45 min. perceptive rehabilitation with proprioceptive components	No intervention	Visual Analogue Scale	↗
IT, 2011 [60]	Age:	55			McGill Pain Rating Index	↗
	Pain area:	LBP			Oswestry Disability Index	=
	Cl. confirmed:	Yes			Wadell Disability Index	=
	Gender (f/m):	54/21				
Paolucci [61]	Total N	45 59	4 weeks, 12×45 min. perceptive treatment with proprioceptive components	3 weeks, 10 sessions (duration per session not reported), Back School based on re-education of breathing, stretching, postural, and strengthening exercises	McGill Pain Questionnaire	=
IT, 2012	Age:	LBP			Centre of Pressure (CoP) area	nA
	Pain area:	Yes			CoP sway length	nA
	Cl. confirmed:	nA			CoP sway velocity AP	nA
	Gender (f/m):				CoP sway velocity LL	nA
Revel [74]	Total N	60	8 weeks, 16×45min. symptomatic analgesics, proprioceptive (head relocation practice), and coordinative (eye/head coordination) exercises, and coordinative exercises	Symptomatic analgesics	Head Repositioning Accuracy (HRA)	↗
FR, 1994	Age:	46.8			Self-reported pain VAS	↗
	Pain area:	NP			Active Range of Motion: Extension	=
	Cl. confirmed:	Yes			Active Range of Motion: Rotation	↗
	Gender (f/m):	51/10			NSAID intake	=
					Self-assessed functional improvement	↗
Sorensen [65]	Total N	207	3 to 9 weeks, 1 to 3 × 30 to 60 min. educational program, stretching	Undefined duration. Symptom based physical training programme, motor control of posture and movement OR therapy ball and dynamic exercises for balance, endurance, and strength	Numeric Pain Rating Scale (NRS)	=
DK, 2012	Age:	39.5			Activity Limitation Scale Fear	=
	Pain area:	LBP			Avoidance Beliefs Questionnaire Back	↙
	Cl. confirmed:	Yes			Beliefs Questionnaire	=
	Gender (f/m):	105/95				
Suni [59]	Total N	106	48 weeks, 96 × 10 exercises for balance, coordination, strength, stretching, motor control, and educational	No intervention (control group)	VAS (past 7 days)	=
FI, 2006	Age:	47.3			ODI	=
	Pain area:	LBP			PDI	=
	Cl. confirmed:	Yes				
	Gender (f/m):	0/100				
Stankovic [68]	Total N	160	4 weeks, 20×30 min. motor control, strengthening, relaxation, breathing, stretching, proprioceptive, and coordinative exercises	4 weeks, 20×30 min. strengthening and stretching aerobic exercises	Oswestry Disability Index (ODI)	↗
RS, 2011	Age:	49.5			ODI subscale Pain	↗
	Pain area:	LBP				
	Cl. confirmed:	No				
	Gender (f/m):	60/140				
Taimela [66]	Total N	76	12 weeks, 12×45min. multimodal treatment: muscle endurance and coordination, relaxation training, educational, motor control, postural control	Neck lectures and activated home exercises (home exercises were introduced and explained in the first two weeks)	Cervical range of motion Cervical	=
FI, 1999	Age:	42.3			Pressure pain threshold	=
	Pain area:	NP			Pain intensity (100mm VAS)	=
	Cl. confirmed:	Yes			Fear Avoidance Beliefs Questionnaire	=
	Gender (f/m):	36/14			Physical impairment in daily activities	=
Taimela [66]	Total N	76	12 weeks, 12×45min. multimodal treatment: muscle endurance and coordination, relaxation training, educational, motor control, postural control	Neck lecture and recommendation of exercises	Cervical range of motion Cervical	=
FI, 1999	Age:	42.3			Pressure pain threshold	=
	Pain area:	NP			Pain intensity (100mm VAS)	↗
	Cl. confirmed:	Yes			Fear Avoidance Beliefs Questionnaire	=
	Gender (f/m):	36/14			Physical impairment in daily activities	=

Legend: LBP = low back pain; NP = neck pain; ↗ in favour of proprioceptive training (PrT); ↙in favour of comparator; = no significant difference. Country codes: AU = Australia; CH = Switzerland; DK = Denmark; FI = Finland; FR = France; IT = Italy; KR = Republic of Korea; NZ = New Zealand; RS = Republic of Serbia; TN = Tunisia; UK = United Kingdom.

The studies involved a total number of N = 1380 subjects with clinically confirmed or self-reported chronic pain persisting for more than three months. Mean symptom duration also varied largely with a range from 8.7 to 328.2 months. The review included 6 studies focussing on neck pain (N = 297) and 12 on LBP (N = 1069). Sample size ranged from 14–207 (mean N = 77 pm 53; 48% females, mean age = 46 pm 8 yrs.). One study did not report age [57], two studies included women [58] or men [59] only.

Most patients were outpatients to the institute carrying out the trial. In one study, the tests were conducted outside the institute at the patients’ workplaces [59]. In most trials the investigator examined the patients for clinical diagnosis. In seven studies, self-report assessments, i.e. pain questionnaires were used for eligibility selection.

### Interventions

Most interventions had patients exercising over a period of 4 to 8 weeks. One study followed patients for one year with measuring events at 6 months and 12 months [59]. Three major directions of PrT were identified. The interventions were described as (1) perceptive PrT (pPrT) where discriminatory perceptive exercises with somatosensory stimuli to the back and joint position sense is practiced [60, 61], (2) as multimodal PrT (mPrT) postural control or balance exercises on labile surfaces often combined with other forms of exercise [59, 62–73], or as (3) head relocation PrT (rPrT) with head-eye coordination exercise [57, 58, 74].

Comparators entailed usual care, home based training, educational therapy, or strengthening, stretching and endurance training. In one study, the intervention was placebo-controlled. The durations of the interventions were between four weeks and 52 weeks (median = six weeks). Table 1 displays an overview of different modalities and dose descriptions.

### Outcomes

Apart from numerical pain rating scales (NRS) and visual analogue scales (VAS), pain outcomes also included the pain subscale from the Oswestry Disability Index (ODI pain), or the McGill Pain Questionnaire; outcome measures on functional status included ODI, the Neck Disability Index (NDI), the Quebec questionnaire and the Roland Morris questionnaire (RMS). For both outcomes, several authors also used self-developed questionnaires (e.g. self-reported functional impairment on non-standardised scales [74]).

Other outcomes assessed range of motion, joint repositioning accuracy, anticipated postural adjustment, and pressure plate posturography. These outcomes were, however, only measured in individual studies and not comparable to other studies within the SR. Furthermore, they were often non-standardised, hence not comparable to studies outside the SR either. For these reasons they were not further evaluated in this SR but included in the overview (Table 1).

### Risk of bias within studies

Arbitration of the third reviewer (EdB) was required for several trials. However, overall inter-rater agreement was found to be substantial with Kappa = 0.73 (p < 0.001, SE = 0.06, 95% CI: 0.62-0.84). Only one trial was deemed free of RoB (Costa et al. [69]). The RoB assessments of all other studies raised some doubt about their results or suggested weakened confidence in the results (Figure 1). Most trials (72%) were rated with a low risk of bias in more than five items of the assessment tool. However, although all studies were registered as RCTs, only 4 trials (22%) clearly reported allocation concealment or use of adequate randomisation procedures. In three studies (17%) the description of blinding suggested high risk of detection bias, as assessor and clinicians appeared to be the same person. Due to study group imbalances at baseline (39%), high dropout rates (34%) and uncontrolled co-interventions (33%) were rated to pose additional high or unclear RoB.

### Risk of bias across studies

Analysis of funnel plots suggested low publication bias in both synthesis of pooled pain and function. See Additional file 4 to view the funnel plots.

### Results of individual studies

Tables 2, 3 and 4 illustrate the synthesised results based on the GRADE considerations described above.

**Table 2:** **Summary of findings of comparison I (perceptual proprioceptive training versus inactive controls or other exercise)**

Patient or population: adults with non-specific chronic low-back pain; Settings: primary and secondary health care centres					
Outcomes	Illustrative means (95% CI)		N (studies)	GRADE	Comments
	Control group	Intervention group			
Comparison 1.1	Inactive control	pPrT			
Pain intensity VAS (0–10) short-term follow-up	The mean pain intensity of the control group was **7.32 points.**	The mean pain intensity in the intervention group was **3.16 points lower** (4.7 to 1.95 lower).	50 (1 study)	++00low^2,3,§^	Significant
Pain intensity VAS (0–10) long-term follow-up	The mean pain intensity of the control group was **7.48 points.**	The mean pain intensity in the intervention group was **3.04 points lower** (4.38 to 1.70 lower).	45 (1 study)	++00low^2,3^	Significant
Back specific functional status ODI short-term follow-up	The mean pain intensity of the control group was **24.32 points.**	The mean ODI score in the intervention group was **4.48 points lower** (11.83 lower to 2.87 higher).	50 (1 study)	++00low^2,3^	Non-significant
Back specific functional status ODI long-term follow-up	The mean pain intensity of the control group was **26.08 points.**	The mean ODI score in the intervention group was **6.38 points lower** (14.98 lower to 2.22 higher).	45 (1 study)	++00low^1,3^	Non-significant
**Comparison 1.2**	**Other exercise**	**pPrT**	**N (studies)**	**GRADE**	**Comments**
Pain intensity various scales short-term follow-up		The mean pain intensity in the intervention group was **1.15 standard deviations lower** (2.93 to 0.63 lower).	80 (2 studies)	000 + very low^2,3,4^	
Pain intensity various scales long-term follow-up	The mean pain intensity of the control group was **4.44 points.**	The mean ODI score in the intervention group was **0.01 points higher** (1.55 lower to 1.57 higher).	45 (1 study)	++00 low^2,3,§^	
Back specific functional status various scales short-term follow-up	The mean ODI score of the control group was **19.04 points.**	The mean ODI score in the intervention group was **0.8 points higher** (5.80 lower to 7.40 higher).	50 (1 study)	++00 low^2,3,§^	
Back specific functional status various scales long-term follow-up	The mean ODI score of the control group was **14.72 points.**	The mean ODI score in the intervention group was **4.98 points higher (2.68 lower to 12.64 higher).**	45(1 study)	++00 low^2,3,§^	

N = total number of patients; CI = Confidence Interval; ^1^Serious limitations in study design (i.e. >25% of participants from studies with high risk of bias); ^2^Serious imprecision (i.e. total number of participants <300 for each outcome or only one study available for comparison); ^3^Indirectness of population (e.g. only one study), intervention (applicability) and outcome measures; ^4^Serious inconsistency (i.e. significant statistical heterogeneity or opposite direction of effects). ^§^Only one study, consistency cannot be evaluated.

GRADE Working Group grades of evidence.

High quality: Further research is very unlikely to change our confidence in the estimate of effect.

Moderate quality: Further research is likely to have an important impact on our confidence in the estimate of effect and may change the estimate.

Low quality: Further research is very likely to have an important impact on our confidence in the estimate of effect and is likely to change the estimate.

Very low quality: We are very uncertain about the estimate.

**Table 3:** **Summary of findings of comparison II (joint repositioning training (rPrT) versus inactive controls or other exercise)**

Patient or population: adults with non-specific chronic low-back pain; Settings: primary and secondary health care centres					
Outcomes	Illustrative means (95% CI)		N (studies)	GRADE	Comments
	Control group	Intervention group			
**Comparison 2.1**	**Inactive control**	**rPrT**			
Pain intensity VAS (0 to 10) scales short-term follow-up	The mean pain intensity ranged across control groups from **4.8 to 7.5 points**	The mean pain intensity in the intervention groups was **1.6 points lower** (3.6 lower to 0.3 higher)	88(2 studies)	+000very low^1,2,4^	
**Comparison 2.2**	**Other exercise**	**rPrT**	**N (studies)**	**GRADE**	**Comments**
Pain intensity Numeric Pain Rating (0–10) short-term follow-up	The mean pain intensity of the control group was reduced by **2.8 points**.	The mean pain intensity in the intervention group was **0.90 points higher** (0.16 lower to 1.96 higher).	58(1 study)	++00 low^2,3,§^	
Back specific functional status Neck Disability Index (0–50) short-term follow-up	The mean NDI score of the control group was reduced by **8.4 points**.	The mean NDI score in the intervention group was **1.50 points higher** (2.06 lower to 5.06 higher).	58 (1 study)	++00 low^2,3,§^	

N = total number of patients; CI = Confidence Interval; ^1^Serious limitations in study design (i.e. >25% of participants from studies with high risk of bias); ^2^Serious imprecision (i.e. total number of participants <300 for each outcome or only one study available for comparison); ^3^Indirectness of population (e.g. only one study), intervention (applicability) and outcome measures; ^4^Serious inconsistency (i.e. significant statistical heterogeneity or opposite direction of effects). ^§^Only one study, consistency cannot be evaluated.

GRADE Working Group grades of evidence.

High quality: Further research is very unlikely to change our confidence in the estimate of effect.

Moderate quality: Further research is likely to have an important impact on our confidence in the estimate of effect and may change the estimate.

Low quality: Further research is very likely to have an important impact on our confidence in the estimate of effect and is likely to change the estimate.

Very low quality: We are very uncertain about the estimate.

**Table 4:** **Summary of findings of comparison III (multimodal proprioceptive Training (mPrT) versus inactive controls, educational approach or other exercise)**

Patient or population: adults with non-specific chronic low-back pain; Settings: primary and secondary health care centres					
Outcomes	Illustrative means (95% CI)		N (studies)	GRADE	Comments
	Control group	Intervention group			
Comparison 3.1	Inactive control	mPrT			
Pain intensity various scales short-term follow-up		The mean pain intensity in the intervention group was **0.55 standard deviations lower** (0.98 to 0.13 lower)	329(4 studies)	+++0^4^moderate	
Pain intensity various scales long-term follow-up		The mean pain intensity in the intervention group was **0.36 standard deviations lower** (0.65 to 0.08 lower)	247(2 studies)	++00^2,4^ low	One additional study did not quantify this outcome but reported no difference between groups.
Back specific functional status various scales short-term follow-up		The mean functional status in the intervention group was **1.39 standard deviations lower** (2.95 lower to 0.16 higher).	246 (2 studies)	++00^2,4^ low	One additional study did not quantify this outcome but reported no difference between groups.
Back specific functional status various scales long-term follow-up		The mean functional status in the intervention group was **0.44 standard deviations lower** (1.80 lower to 0.92 higher).	246 (2 studies)	+++0^2^ moderate	One additional study did not quantify this outcome but reported no difference between groups.
**Comparison 3.2**	**Other exercise**	**mPrT**	**N (studies)**	**GRADE**	**Comments**
Pain intensity various scales short-term follow-up		The mean pain intensity in the intervention group was **0.40 standard deviations lower** (0.84 lower to 0.05 higher)	465 (8 studies)	++00^2,4^ low	
Pain intensity various scales long-term follow-up	The mean pain intensity of the control group was **35.7 points.**	The mean pain intensity in the intervention group of one study was **13.4 points** higher (5.96 to 20.84 higher).	122 (1 studies)	++00^2,4^ low	One additional study did not quantify this outcome but reported no difference between groups.
Back specific functional status various scales short-term follow-up		The mean pain intensity in the intervention group was **0.45 standard deviations lower** (0.83 to 0.08 lower)	466 (8 studies)	++00^2,4^ low	One additional study did not quantify this outcome but reported no difference between groups.
Back specific functional status various scales long-term follow-up	The mean pain intensity of the control group was **16.2 points.**	The mean pain intensity in the intervention group of one study was **3.2 points** higher (1.55 lower to 7.95 higher).	107 (1 studies)	++00^2,3^ low	One additional study did not quantify this outcome but reported no difference between groups.
**Comparison 3.3**	**Educational approach**	**mPrT**	**N (studies)**	**GRADE**	**Comments**
Pain intensity VAS scales (0–10) short-term follow-up	The mean pain intensity of the control group was **4.9 points.**	The mean pain intensity in the intervention group was **0.30 points higher** (0.32 lower to 0.92 higher).	185 (1 study)	++00^2,3,§^ low	
Pain intensity various scales long-term follow-up	The mean pain intensity of the control group was **4.5 points.**	The mean pain intensity in the intervention group was **0.30 points higher** (0.40 lower to 1.00 higher).	164 (1 study)	++00^2,3,§^ low	
Back specific functional status LBP rating scale short-term follow-up	The mean score on the LBP rating scale of the control group was **11.6 points.**	The mean pain intensity in the intervention group was **1.40 points higher** (0.33 lower to 3.13 higher).	185 (1 study)	++00^2,3,§^ low	
Back specific functional status LBP rating scale long-term follow-up	The mean score on the LBP rating scale of the control group was **11.0 points.**	The mean pain intensity in the intervention group was **2.00 points higher** (0.06 to 3.94 higher).	164 (1 study)	++00^2,3,§^ low	

N = total number of patients; CI = Confidence Interval; ^1^Serious limitations in study design (i.e. >25% of participants from studies with high risk of bias); ^2^Serious imprecision (i.e. total number of participants <300 for each outcome or only one study available for comparison); ^3^Indirectness of population (e.g. only one study), intervention (applicability) and outcome measures; ^4^Serious inconsistency (i.e. significant statistical heterogeneity or opposite direction of effects). ^§^Only one study, consistency cannot be evaluated.

GRADE Working Group grades of evidence.

High quality: Further research is very unlikely to change our confidence in the estimate of effect.

Moderate quality: Further research is likely to have an important impact on our confidence in the estimate of effect and may change the estimate.

Low quality: Further research is very likely to have an important impact on our confidence in the estimate of effect and is likely to change the estimate.

Very low quality: We are very uncertain about the estimate.

#### Comparisons I: pPrT versus other exercises or inactivity

Two studies, one with a low risk of bias [60] the other with high risk of bias [61], compared pPrT with other exercises. In both studies, the exercise group also received back education as part of the control intervention (Table 2). Both studies evaluated pain intensity as an outcome, although only one recorded long-term follow-up results [60]. The pooled SMD (95% CI) between groups was −1.15 (−2.93 to 0.63) in the short-term. The follow-up results of the long-term RCT showed no significant difference between back school exercises and pPrT groups (N = 45). There is very low quality evidence that pPrT is more effective for pain reduction than back school exercise in the short-term (two RCTs; N = 80; limitations in design, imprecision, inconsistency).

The RCT with low RoB [60] additionally compared pPrT to an inactive control group. The pain score was significantly lower in the pPrT group than in the inactive control group at the end of the treatment (N = 50) and at the long-term follow-up (N = 45). Study outcomes also included a back specific functional status, assessed with the Oswestry Disability Index. No significant group differences were found at short- (N = 45) or long-term follow-up (N = 50) for this outcome. With only one RCT and limitations in imprecision and indirectness (due to applicability of intervention and small total sample size) there is low quality evidence that there is no significant difference in effect on functional status between pPrT and not intervening at all. Further, there is only low quality evidence that pPrT is more effective for pain rehabilitation when compared to inactive controls.

#### Comparisons II: rPrT versus other exercises or inactivity

Two studies with high risk of bias showed significant group interactions for self-reported pain in favour of the rPrT intervention [57, 74] (Table 3). Both compared change of VAS after head-eye coordination exercises with an inactive group of patients with chronic neck pain (MD (95% CI) = −1.6 (−3.6 to 0.3). Co-interventions were not controlled. There is very low evidence (2 RCTs; N = 103; limitations in design, imprecision, and inconsistency) that rPrT is more effective in short-term reduction of pain than not intervening at all.

One study with low RoB [58] compared a 6-week proprioceptive head-eye coordination program with conventional physiotherapy without PrT elements but found no group differences at 6 weeks follow-up. There is low quality evidence (1 RCT; N = 58; limitations in imprecision and indirectness) that there is no difference in short-term effectiveness of rPrT on self-reported pain compared to other exercises.

The same RCT [58] compared rPrT to stretching and strengthening exercises and found no group differences on the neck specific functional status using the Neck Disability Index after a 6-week intervention period. There is low evidence (1 RCT, N = 58; limitations in imprecision and indirectness) that there is no difference in short-term improvement on functional status between rPrT and other forms of exercise.

#### Comparisons III: mPrT versus other exercises, inactivity, or behavioural approach

Four studies compared mPrT effects on pain to inactive control groups [59, 66, 69, 71] (Table 4). The Taimela study (low RoB) found significant reduction of neck pain [66] immediately after a 12-week multimodal intervention period, but not at the one-year follow-up measurement. However, as this study did not quantify the long-term follow-up of its outcomes on pain and function, it was not included in the synthesis of results. Two other mPrT studies (low RoB) found significant group differences at long-term follow-up after one year [59, 69], but no short-term differences. Only one mPrT study was not biased by co-interventions [71] but had other limitation (sample size and baseline imbalances). Otherwise low in RoB, the study described significant reduction of neck pain after 5 weeks of mPrT whereas pain persisted in the non-exercise control group. There is moderate quality evidence that a multimodal intervention with proprioceptive elements is more effective on pain alleviation at post-treatment than not intervening at all (4 RCTs, N = 329; limitations in inconsistency). There is low quality evidence (2 RTCs, n = 247; limitations in imprecision and inconsistency) on the effectiveness of mPrT compared to inactive control groups on self-reported pain at long-term follow-up.

The Costa study with low RoB showed significant group differences for the RMS functional scale [69] when compared to the placebo control group after the 8-week therapy program. One low RoB study reported no significant group differences for functional status outcomes [59]. The pooled SMD (95% CI) between groups was −1.39 (−2.95 to 0.16). There is low quality evidence (2 RCTs, n = 246; limitations in imprecision and inconsistency) that mPrT is more effective compared to inactive or placebo control groups on functional status of LBP patients at short-term assessment. There is moderate quality evidence (2 RCTs, n = 229; limitations in imprecision) that mPrT is no more effective compared to inactive controls at long-term follow up.

Eight RCTs compared the effects of mPrT with other forms of active treatments and exercises. Significant between group differences in favour of mPrT was found in two high RoB studies immediately after a four-week intervention [68]. Two further studies with high RoB reported significant pain reduction [64, 73] but no more than when the same exercises were performed without additional PrT-elements. The latter findings were confirmed by three low RoB studies where no group differences are reported [62, 63, 66]. One high RoB study [67] reported significant group differences in favour of the control group with no PrT elements. There is low quality evidence (8 RCTs; N = 465 and 122 for short- and long-term respectively; limitations in design and inconsistency) that mPrT is more effective than other exercise interventions on reduction of self-reported pain (short or long-term). Comparison of various back specific functional scales showed short-term effects with significant group difference in one study with low RoB [63] and in two further studies with high RoB [68, 73]. There is low quality evidence (8 RCTs; N = 466 and 1 RCT with N = 107 for short- and long-term respectively; limitations in imprecision and indirectness) on the effectiveness of mPrT on functional restoration.

Sorensen et al. [65] tested an educational approach against symptom-based physical training with PrT elements. Similar improvements were reported after the 8-week intervention period with no long-term improvement in either one of the groups. There is low quality evidence (1 RCT, N = 185 and N = 164 for short- and long-term respectively; limitations in imprecision and indirectness) that mPrT is no more effective for pain alleviation when compared to an educational method (short or long-term follow-up). Comparison of functional outcomes [65, 66] showed no group differences at short- or long-term assessments. There is low quality evidence (1 RCT, N = 185; limitations in imprecision and indirectness) that mPrT is similarly effective as an educational approach to functional restoration of patients with neck or low back pain. There is low quality evidence that (1 RCT; N = 164; limitations in imprecision and indirectness) that mPrT is less effective for long-term treatment of NSLBP than the educational approach.

## Discussion

This SR attempted to provide an overview of current evidence for the use of PrT in rehabilitation of patients with chronic neck- and back pain. Its secondary aim was to identify practical features of PrT strategies that resulted in positive outcomes, i.e. alleviating self-reported pain and improved functional status. The collected data from 18 studies after an extensive search in all relevant databases suggest that no conclusive evidence exists to support the implementation of PrT interventions in back- or neck-pain rehabilitation. On the other hand, most interventions with PrT elements did report some reduction in pain and improvement of functional status, but the methodological approaches do not allow drawing an arrow of causality to either the PrT intervention or defective neuromuscular signalling. With multiple low-quality RCTs reporting conflicting findings on the effectiveness of PrT on pain and functional status, this qualitative analysis cannot provide any conclusive recommendations.

### Methodological limitations of included studies

The overall quality of the studies was low and RoB assessment revealed considerate methodological shortcomings posing moderate to high risk of bias. Such findings cannot be ignored, particularly in research on subjective outcomes such as pain and functional status [75]. Strong empirical evidence suggests that such violations of fundamental methodological guidelines, e.g. failure to conceal allocation sequence in randomized trials, is associated with overestimation of effects [76]. Solidly performed randomisation allows for the sequence to be unpredictable [75] and if assignments are non-random, deciphering of sequence can occur. Missing outcome data, due to attrition during the study or exclusion from the analysis was apparent in many included reports and may have led to overestimation of effects [75]. A further source of bias often found was baseline imbalance, which might suggest bias in allocation and could cause statistical bias. Thus, differences in outcomes could be due to characteristics of patients rather than treatment [77]. Similarly, it was observed that most studies did not measure proprioceptive outcomes hence diminishing the conclusion to make any connection of the experienced effect on proprioceptive signalling or neuromuscular control [60]. To properly understand the effects of PrT on pain and function, proprioception itself should also be observed, preferably using neurophysiological measurements (e.g. proprioceptive evoked potential [78]). In light of these methodological shortcomings, it is not possible to substantiate or refute the assumption of the superiority of PrT rehabilitation over other approaches.

### Recommendations on PrT implementation

Apart from the many definitions of PrT, there are no recommendations or practical cornerstones of an effective PrT. In any exercise, proprioception and other sensory inputs are involved [43, 79]. Moreover, frequency, dosage, and duration are other factors applied in a variable way. Inconsistent use of exercise protocols might lead to potential intervention bias regarding the evidence of optimal training protocols to be used in non-specific musculoskeletal pain [75]. Sample sizes of future trials should be large enough to enable sub group and dose–response analyses. With no standardised procedure of PrT it is impossible to create effective pooling of outcome data. The question on how long PrT would have to be exercised or how often it should be done (e.g. on a daily basis, once every week) and at which intensity cannot be answered in this review.

### Limitations

The RoB rating proved to be challenging and relatively high inconsistency between the review authors in one particular item (selective reporting) was apparent. Using standardised scales for rating methodological quality leads to some practical issues. Blinding of therapists and patients is often not possible where the intervention is as obvious as is PrT. The assessment tool by Cochrane addresses this issue in a pragmatic way by allowing reviewers to assess importance of each item and rate level of risk in the context of the field of research. This is at the same time the tool’s greatest weakness, as it does not delimit the scale with clear boarders. This may cause incongruences between review authors with different levels of methodological training or content knowledge [55]. Lack of elaborations and clarity in described methods also contributed to the difficulties while rating the quality of the studies. Hence, allocation procedure and sequence generation could not be derived from the provided information in the text. Although several authors were contacted for this reason, the missing information could not be obtained. This lack in reporting quality should be addressed in future studies by explicitly referring to international guidelines, such as the CONSORT statements [76].

Language bias might have led to the exclusion of important findings. One study from Poland and one from Iran (both RCTs) had to be excluded, as no English full texts were available [80, 81].

Meta-analysis could not be conducted on all comparisons and outcome measures due to the methodological and statistical heterogeneity. The attempt to reduce heterogeneity through selected analysis of two further subgroups based on outcomes (e.g. VAS and NRS) and population (neck and back pain) had no effect. Subgroups were clinically still very different from each other, e.g. comparing back pain population receiving perceptive rehabilitation with neck pain population receiving joint repositioning exercise (e.g. [60] vs. [58]). Furthermore, due to the previously mentioned lack of reporting quality, there were insufficient data to report all relevant outcomes required for accurate meta-analyses.

A further limitation of the review was delimiting the included interventions. Because of the arbitrary use of expressions (cybernetic exercise, sensorimotor training, etc.), it cannot be guaranteed that all studies addressing PrT were included. There is no consistent term for it. In this sense it may be argued that motor control exercises [69] and perceptive rehabilitation [60] should not have been included in this SR, or, conversely, Saner et al., who assessed movement control exercises in a RCT [82], should have been included. This, however, is one of the reasons it has become so important to conduct a SR on the topic: to collect the existing information, summarise the evidence, and allow practitioners explain the rational of their interventions. Clearly defining the population and intervention of SR is always difficult in rehabilitation research [52]. The challenge of this particular topic is that it tries to connect two opaque phenomena not fully understood. Sensorimotor changes on spinal and supra-spinal level are subject to on-going debates and it is not entirely clear what actually happens on cortical levels when pain becomes chronic [83] and movement behaviour changes [84]. Pain is a complex phenomenon, which, for practical reasons, is often recorded with subjective outcome measures [85] and is not always related to functional impairment. The population included may have a variety of different causes for their pain; hence, function will not necessarily improve when pain does [86]. Verra et al. and Luomajoki et al. have shown how subgroups of fibromyalgia and LBP patients may exist and could respond differently to treatments [87, 88]. Thus, sample sizes of future trials should be large enough to enable subgroups in order to compare NSLBP patients with and without sensorimotor deficiencies. To allow comprehensive and evidence based recommendations for the implementation of sensorimotor exercises (i.e. PrT) there is still need for large scale, high quality RCTs including dose–response analyses based on objective outcome measures of physiological change.

## Conclusions

There are not enough interventions conducted in a methodologically solid way to make any conclusive statements on the effects of PrT on pain and function in patients with chronic neck- or LBP. The included studies suggest a tendency towards demonstrable benefits from the PrT intervention, particularly for functional outcomes. Moreover, there is low quality evidence that PrT adds no benefits to conventional therapy. However, findings are inconsistent among different studies. There is low quality evidence that PrT is inferior to educational approaches, which aim at change in behaviour and attitude. Based on the reviewed studies, no recommendations on PrT mode and implementation can be given.

Future research on the effect of PrT should try to compare more generalizable samples and clearly define the framework of PrT. Efforts towards a standardised PrT should involve practical experience and incorporate the evidence of basic neurophysiological research. Interventions have to be reported with more care to important details to allow comparison, e.g. group allocation and the definition of proprioception.

## Electronic supplementary material

Additional file 1:Review Protocol. Extract of the protocol with a-priori defined research questions, search strategy, analyses, and eligibility criteria. (PDF 106 KB)

Additional file 2:Eligibility Criteria. Final eligibility criteria used for study selection. (PDF 62 KB)

Additional file 3:Example search. Example search (Medline). (PDF 23 KB)

Additional file 4:Funnel plots.(ZIP 12 KB)

Below are the links to the authors’ original submitted files for images.

Authors’ original file for figure 1

Authors’ original file for figure 2
