# Supplementary material for: Effects of proprioceptive exercises on pain and function in chronic neck- and low back pain rehabilitation: a systematic literature review
Source: BMC Musculoskelet Disord. 2014 Nov 19;15:382. doi: 10.1186/1471-2474-15-382 (PMC4247630; doi:10.1186/1471-2474-15-382)
Supplement: Supplementary file 1 — Additional file 1:Review Protocol. Extract of the protocol with a-priori defined research questions, search strategy, analyses, and eligibility criteria. (PDF 106 KB) [file 12891_2014_2326_MOESM1_ESM.pdf]

What is the research question?

Table 1: PICO structured research question

Free form questions	**P**opulation	**I**ntervention	**C**omparator	**O**utcome
Do sensorimotor training concepts reduce pain in adult patients with non-specific musculoskeletal pain and improve functionality more effectively than conventional therapy?	Adult non-specific musculoskeletal pain patients	Sensorimotor training concepts	Conventional therapy	Pain and quality of life related & measures of functionality
Do sensorimotor training concepts reduce pain in adult patients with non-specific musculoskeletal pain and improve functionality more effectively than conventional therapy?	Adult non-specific musculoskeletal pain patients	Sensorimotor training concepts	No intervention	Pain and quality of life related & measures of functionality
What are the features of an effective sensorimotor training that reduces pain and improves functionality in patients with non-specific musculoskeletal pain?	Adult non-specific musculoskeletal pain patients	Sensorimotor training concepts	Ineffective sensorimotor training concepts	Features of the intervention method applied

What is the search strategy?

We will apply the methods recommended by the Cochrane Effective Practice and Organisation of Care (EPOC) group [1]. A life and health sciences librarian shall assist the trials search coordinator in the development of the search strategy. The following databases will be searched for primary literature (from inception to present): COCHRANE, CENTRAL, MEDLINE, EMBASE, CINAHL, DARE

Search terms will be defined for the population, intervention, and outcome and combined using the Boolean logic operators AND, OR and NOT.

Free search: (perceptive OR proprioceptive OR sensorimotor OR sensorymotor OR sensory-motor) AND ((nonspecific AND pain) OR (musculoskeletal AND pain))

A list of potentially relevant references found through the above search strategy, including grey literature, will be exported to a tabular separated table for first identification of irrelevant references (according to criteria described below). Once recorded, references will remain in the table and categorised according to its status in the review process: Included, Excluded, Ongoing Study, or Pending.

What are the inclusion/exclusion criteria?

Titles and abstracts, that will be retrieved from the previously describes electronic search, will be screened by the first two authors (see appendix for flow chart). The inclusion criteria are described as follows:

Participants: Adult patients with chronic (pain persists for more than three months) or recurring non-specific musculoskeletal pain including whiplash-associated disorders. Using the expression 'recurring' is aimed at including studies investigating patients who suffer from frequently occurring pain in the same region, but which usually subdues without external intervention. Only studies declaring clinical examination or interview assessment for neurological deficits related to peripheral or central nerve damage, vestibular diseases, osteo-articular diseases (e.g. rheumatoid arthritis), fractures, and tumours, and clearly stating the absence of any of these conditions, will be included. No restrictions regarding sex, ethnicity, language, or clinical setting (inpatients or outpatients) will be made.

Intervention: All variations of sensorimotor training (all training types that address the sensory system as well as motor control, e.g. training on a labile surface or dual task exercise) compared to methods not including the sensory motor system (e.g. massage) or to no intervention at all.

Outcomes: As there is no gold standard to measure the construct under investigation, many different ways are being used to determine the functional status and pain by different studies. Depending on the aim of the study, the primary outcome might vary greatly. Generally, eligible studies should include outcomes addressing pain perception, activity, function, or participation, e.g. [2, 3]:

- Back Pain Functional Scale
- Numeric Pain Rating Scale (NPRS)
- Oswestry Low Back Pain Disability Questionnaire
- Roland-Morris Questionnaire
- Visual Analogue Scale for Pain (VAS)
- Quality of life related measures
- Range of motion and other activity related measures etc.)
- Measure of life Scale for P

Studies: Randomised controlled trials (RCTs), quasi-RCTs, and controlled before and after studies (CBAs).

How will the data be extracted and analysed?

A health science librarian and MMc will individually apply the search strategy to produce the initial data set. Together, with one co-author (CSch), but independently, MMc will sequentially screen titles, abstracts, and full texts for inclusion. Inter-rater agreement will be assessed using percentage agreement and Cohen's Kappa statistic [4]. Agreement is interpreted in accordance with Landis and Koch's benchmarks for assessing the agreement between raters: poor (<0), slight (.0-.20), fair (.21-.40), moderate (.41-.60), substantial (.61-.80), and almost perfect (.81-1.0) [5]. Titles will be screened for PICOS described above, however, no language restrictions will be made. All abstracts that will be selected by either one of the reviewers will be retrieved from the database. Thereby selected

abstracts will again be screened for exclusion criteria not revealed in titles. Full text articles for all included studies will be acquired by all possible means, if available.

If the methods described in the study's full text involves sensorimotor training methods, if at least one of pain or quality of life was measured as an outcome, and if the previous described criteria are confirmed, the study will be included for data analysis.

Any disagreement is to be resolved by discussion between the review authors and arbitrator (EdB) as required.

To reduce risk of bias and provide interpretable data, recommendations provided by Cochrane Pain group [6] and PRISMA guidelines [7] are consulted for data extraction. Both review authors (MMc and CSch) will individually extract data and record on a standardised data-extraction form based on the template by the Cochrane Pain group [8]. Data will be entered directly into a tabular separated form (e.g. Microsoft Excel) to allow subsequent data analysis with standard statistical software (e.g. R Studio or Matlab).

The extracted data should include study design and methodology (including randomisation procedures and settings), participants' characteristics, details of the intervention(s), dropouts and withdrawals, and outcome measures at baseline and endpoint.

In case of too high heterogeneity (e.g. $I^2 > 40\%$, or Cochrane's Q p-value < 0.1), data will be synthesized qualitatively using levels of evidence as suggested by Tudler et al. from Cochrane Collaboration Back Review group [11].

If outcomes can be standardized to a common pain and quality of life outcome, and if participants prove to be homogeneous, quantitative effect size statistic will be applied to establish whether there is evidence of an effect, estimate the size of the effect, and investigate whether the effect is consistent across studies.

References:

1. **Cochrane Handbook for Systematic Reviews of Interventions** [http://www.cochrane-handbook.org]
2. Oesch P: **Assessments in der muskuloskelettalen Rehabilitation**. Bern: Huber-Hans Verlag; 2007.
3. Finch E: **Physical Rehabilitation Outcome Measures: A Guide to Enhanced Clinical Decision Making**: B. C. Decker Incorporated; 2002.
4. Cohen A: **Comparison of correlated correlations**. *Statistics in medicine* 1989, **8**(12):1485-1495.
5. Koch GG, Landis JR, Freeman JL, Freeman DH, Jr., Lehnen RC: **A general methodology for the analysis of experiments with repeated measurement of categorical data**. *Biometrics* 1977, **33**(1):133-158.
6. **Authoring or assessing a Cochrane Protocol, Review, or Review Update for the PaPaS Review Group**. In. Oxford: Cochrane Pain, Palliative & Supportive Care Review Group; 2012.
7. Liberati A, Altman DG, Tetzlaff J, Mulrow C, Gotzsche PC, Ioannidis JP, Clarke M, Devereaux PJ, Kleijnen J, Moher D: **The PRISMA statement for reporting systematic reviews and meta-analyses of studies that evaluate healthcare interventions: explanation and elaboration**. *BMJ* 2009, **339**:b2700.
8. Hollis S, Leonard T. In. Oxford: Cochrane Pain, Palliative & Supportive Care Review Group; 2011.

9. Gavaghan DJ, Moore RA, McQuay HJ: **An evaluation of homogeneity tests in meta-analyses in pain using simulations of individual patient data**. *Pain* 2000, **85**(3):415-424.
10. Ioannidis JP, Patsopoulos NA, Evangelou E: **Uncertainty in heterogeneity estimates in meta-analyses**. *BMJ* 2007, **335**(7626):914-916.
11. van Tulder M, Furlan A, Bombardier C, Bouter L: **Updated method guidelines for systematic reviews in the cochrane collaboration back review group**. *Spine (Phila Pa 1976)* 2003, **28**(12):1290-1299.
