# Supplementary material for: Effects of proprioceptive exercises on pain and function in chronic neck- and low back pain rehabilitation: a systematic literature review
Source: BMC Musculoskelet Disord. 2014 Nov 19;15:382. doi: 10.1186/1471-2474-15-382 (PMC4247630; doi:10.1186/1471-2474-15-382)
Supplement: Supplementary file 2 — Additional file 2:Eligibility Criteria. Final eligibility criteria used for study selection. (PDF 62 KB) [file 12891_2014_2326_MOESM2_ESM.pdf]

Amended eligibility criteria

	Inclusion	Exclusion
Population	Adult patients with musculoskeletal low back- or neck pain	- Peripheral or central nerve damage<br>- Vestibular disease<br>- Osteo-articular diseases (e.g. rheumatoid arthritis)<br>- Prostheses<br>- Fractures<br>- Carcinogenic pain<br>- Ankle instability alone<br>- Osteoporosis<br>- Complex Regional Pain Syndrome<br>- Head ache alone<br>- Mandibular pain/Myofascial pain<br>- Fibromyalgia<br>- Patellofemoral pain syndrome.<br>- Shoulder pain alone<br>- Pelvic pain alone
Intervention	Proprioceptive Training:<br>Training methods must meet ALL the following features:<br>- Slow **closed-loop** control of movement involving efferent as well as afferent information processing (e.g. single leg stance or walking on uneven surface)<br>- **Changing task** goals (adjustable difficulty)<br>- **Dual task** to overcome limited attentional capacity<br>(as defined by Kim D, Van Ryssegem G, Hong J: **Overcoming the Myth of Proprioceptive Training**. *Clinical Kinesiology (Spring)* 2011, **65**(1):18-28.	- No intervention<br>Manual therapy alone<br>- Passive therapy alone (e.g. massage, thermal, fango, taping, bracing, whole body vibration, chiropractice)<br>- Drug intervention<br>- work related exercises (added<br>- PNF: proprioceptive neuromuscular facilitation<br>- Yoga<br>- Psychological treatment alone<br>- Educational treatment alone (e.g. back school without exercise)<br>- Global Postural Re-Education<br>- Pilates
Outcome	Pain OR/AND functional status	No pain/no functional status
Study Design	Randomized Control Trial	Comparative, cross-sectional studies<br>uncontrolled studies<br>case-control studies<br>Qualitative studies
