# Supplementary material for: Effects of proprioceptive exercises on pain and function in chronic neck- and low back pain rehabilitation: a systematic literature review
Source: BMC Musculoskelet Disord. 2014 Nov 19;15:382. doi: 10.1186/1471-2474-15-382 (PMC4247630; doi:10.1186/1471-2474-15-382)
Supplement: Supplementary file 3 — Additional file 3:Example search. Example search (Medline). (PDF 23 KB) [file 12891_2014_2326_MOESM3_ESM.pdf]

Database(s): **Ovid MEDLINE(R), Ovid MEDLINE(R) In-Process & Other Non-Indexed Citations, Ovid MEDLINE(R) Daily and Ovid OLDMEDLINE(R)** 1946 to Present

Search Strategy:

#	Searches	Results
1	exp musculoskeletal pain/ or back pain/ or low back pain/ or chronic pain/ or piriformis muscle syndrome/ or nociceptive pain/ or pain, intractable/ or pelvic pain/	36492
2	Patellofemoral Pain Syndrome/	390
3	Joint Instability/	13799
4	Reflex Sympathetic Dystrophy/	3073
5	Shoulder Pain/	2520
6	Myofascial Pain Syndromes/	1141
7	Fibromyalgia/	5692
8	or/1-7	61816
9	((pain or ache or discomfort or trouble or hurt or "muscle imbalance" or "muscular imbalance" or "muscle stiffness" or "muscle tightness") adj5 (shoulder or back or hip or "lower limb" or "upper limb" or neck or pelvic or muscular or muscle or joint or cervical or lumbar or refractory or "patellofemoral syndrome" or musculoskeletal or myofascial or "joint instability" or "piriformis muscle syndrome" or fibromyalgia)).ti,ab.	69953
10	8 or 9	108153
11	exp Arthritis/	192690
12	exp Spinal Cord Injuries/	35407
13	exp Arthroplasty, Replacement, Hip/	15480
14	exp Arthroplasty, Replacement, Knee/	11051
15	exp Rheumatic Diseases/	168303
16	Intervertebral Disc Displacement/	15191
17	Anterior Cruciate Ligament/	8948
18	exp Rupture/	33821
19	Shoulder Impingement Syndrome/ or Femoracetabular Impingement/	1349

20	(arthritis or arthrosis or "spinal cord injury" or tumor or tumour or cancer or neoplas* or "total knee replacement" or "total hip replacement" or arthroplasty or rheuma or "slipped disc" or hernia* or "anterior cruciate ligament" or rupture or impingement).ti,ab.	1900693
21	or/11-20	2060593
22	10 not 21	74650
23	((sensorimotor or "sensory motor" or sensory-motor or proprioception or proprioceptive or balance or "postural balance" or posture or postural or coordination or "motor coordination" or coordinative or cybernetic or multisensory or stabilizing or stabilising) adj15 (therap* or remad* or exercise* or train* or rehabilitation or technique*)).ti,ab.	16769
24	Feedback, Sensory/ or Postural Balance/	13437
25	cybernetics/	2116
26	Proprioception/	5853
27	24 or 25 or 26	20492
28	(therap* or remad* or exercise* or train* or rehabilitation or technique*).ti,ab.	2997152
29	27 and 28	4770
30	23 or 29	19380
31	22 and 30	776
32	RANDOMIZED CONTROLLED TRIAL.pt.	343002
33	CONTROLLED CLINICAL TRIAL.pt.	85733
34	RANDOMIZED CONTROLLED TRIAL.sh.	343002
35	RANDOM ALLOCATION.sh.	76622
36	DOUBLE BLIND METHOD.sh.	118556
37	SINGLE BLIND METHOD.sh.	17105
38	or/32-37	499216
39	(ANIMALS not HUMANS).sh.	3721536
40	38 not 39	455904
41	CLINICAL TRIAL.pt.	476906
42	exp clinical trial/	707267
43	(clin$ adj25 trial$).ti,ab.	234754
44	((singl$ or doubl$ or trebl$ or tripl$) adj25 (blind$ or mask$)).ti,ab.	124598
45	PLACEBOS.sh.	31597

46	placebo$.ti,ab.	147069
47	random$.ti,ab.	632437
48	RESEARCH DESIGN.sh.	68723
49	or/41-48	1285759
50	49 not 39	1190446
51	50 not 40	750481
52	COMPARATIVE STUDY.sh.	1622202
53	exp EVALUATION STUDIES/	175855
54	FOLLOW UP STUDIES.sh.	463889
55	PROSPECTIVE STUDIES.sh.	334413
56	(control$ or prospectiv$ or volunteer$).ti,ab.	2771199
57	or/52-56	4487554
58	57 not 39	3521294
59	58 not (40 or 51)	2853900
60	40 or 51 or 59	4060285
61	intervention studies/	5705
62	(intervention* adj3 (stud* or trial or setting or procedure)).mp. [mp=title, abstract, original title, name of substance word, subject heading word, protocol supplementary concept, rare disease supplementary concept, unique identifier]	35935
63	61 or 62	35935
64	60 or 63	4071640
65	31 and 64	472
66	limit 31 to ("review" or systematic reviews)	168

1. **Manual therapy with and without vestibular rehabilitation for cervicogenic dizziness: a systematic review.**

   Lystad RP. Bell G. Bonnevie-Svendsen M. Carter CV.

   Chiropractic & manual therapies. 19(1):21, 2011.
   [Journal Article]

   UI: 21923933

Authors Full Name

Lystad, Reidar P. Bell, Gregory. Bonnevie-Svendsen, Martin. Carter, Catherine V.
