# Supplementary figures and images for: Effects of proprioceptive exercises on pain and function in chronic neck- and low back pain rehabilitation: a systematic literature review
Source: BMC Musculoskelet Disord. 2014 Nov 19;15:382. doi: 10.1186/1471-2474-15-382 (PMC4247630; doi:10.1186/1471-2474-15-382)

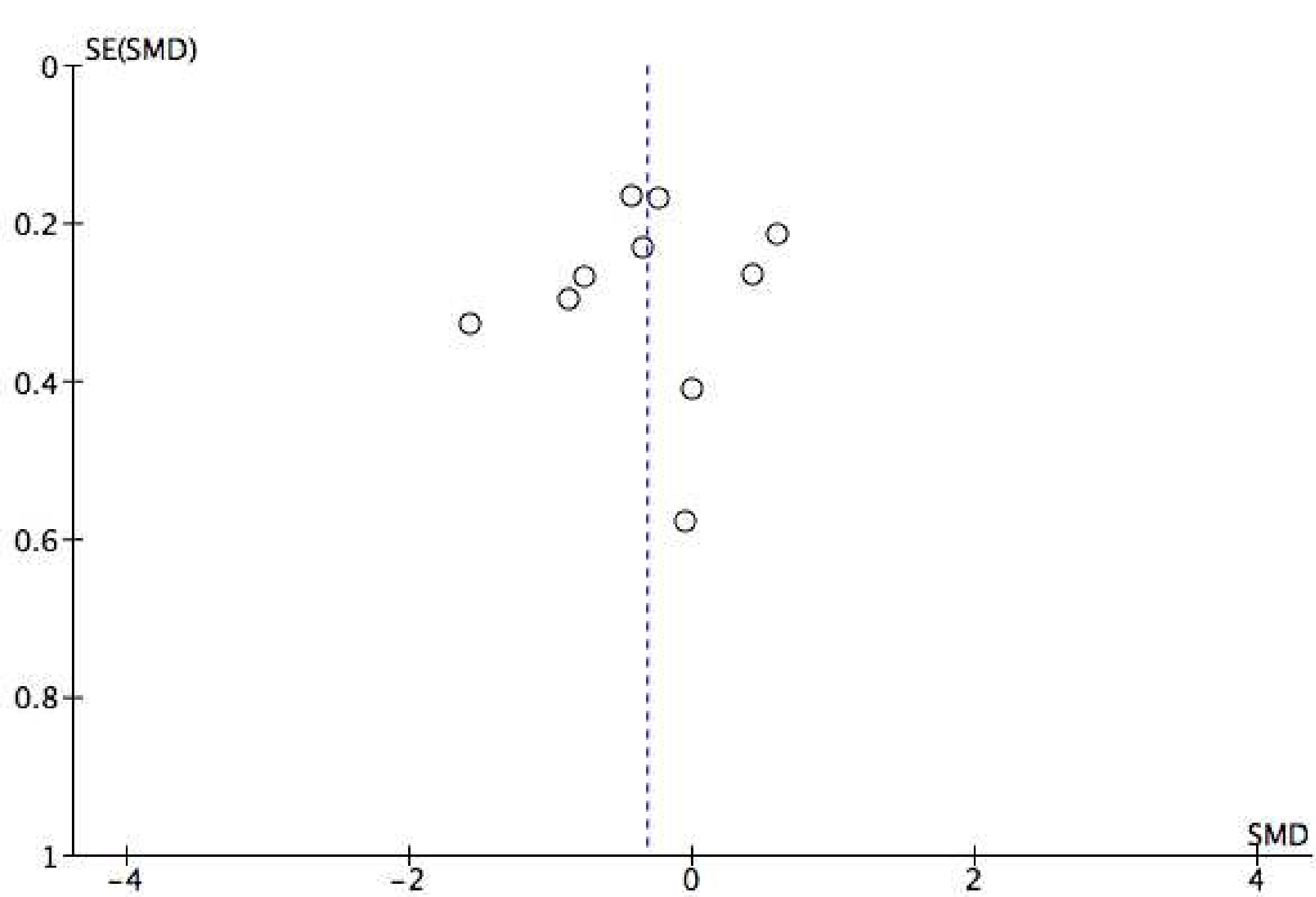

Supplement: Supplementary file 4 — Additional file 4:Funnel plots.(ZIP 12 KB) [file 12891_2014_2326_MOESM4_ESM.zip › 2127348251437560_add4.pdf]

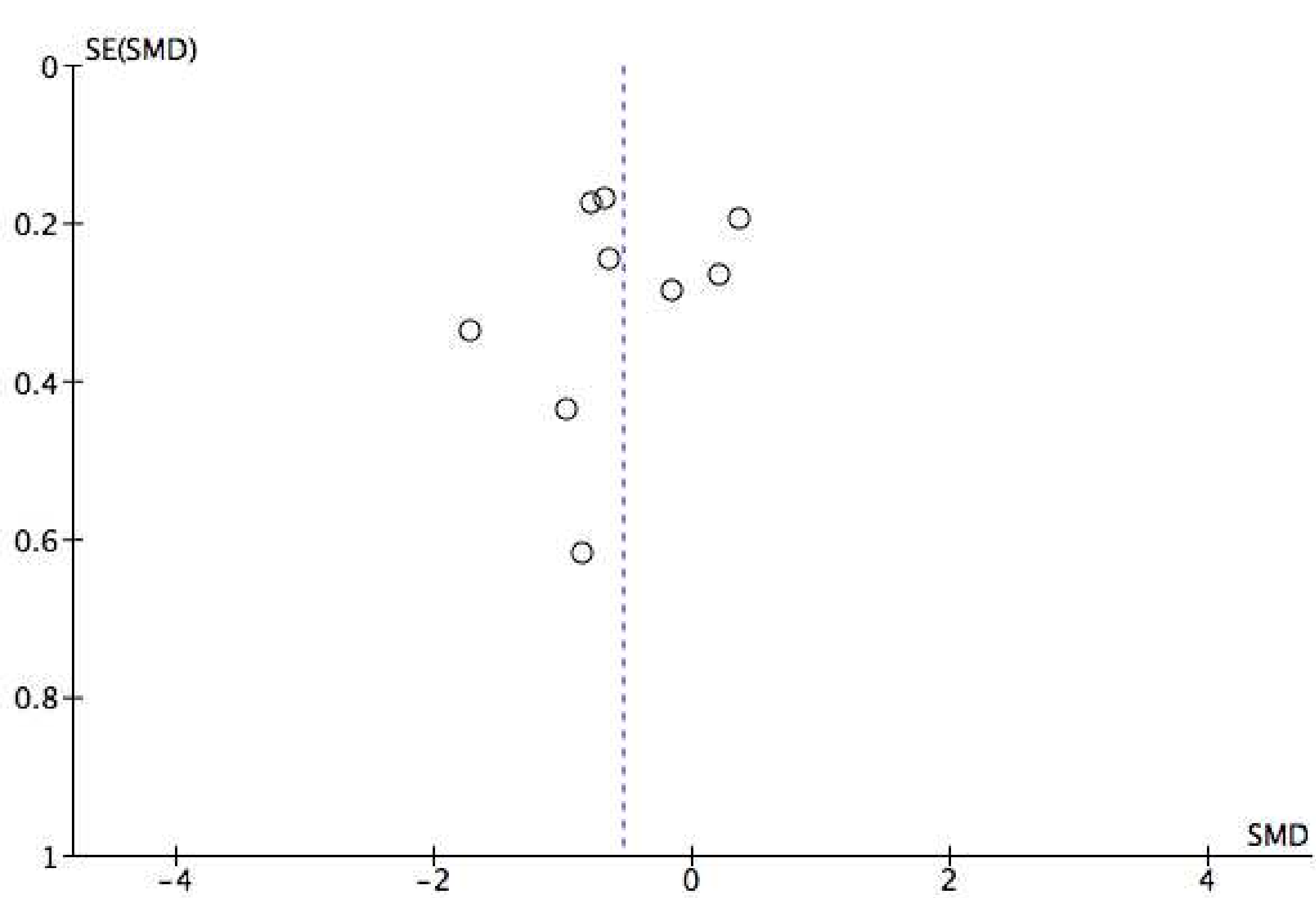

Supplement: Supplementary file 4 — Additional file 4:Funnel plots.(ZIP 12 KB) [file 12891_2014_2326_MOESM4_ESM.zip › 2127348251437560_add5.pdf]

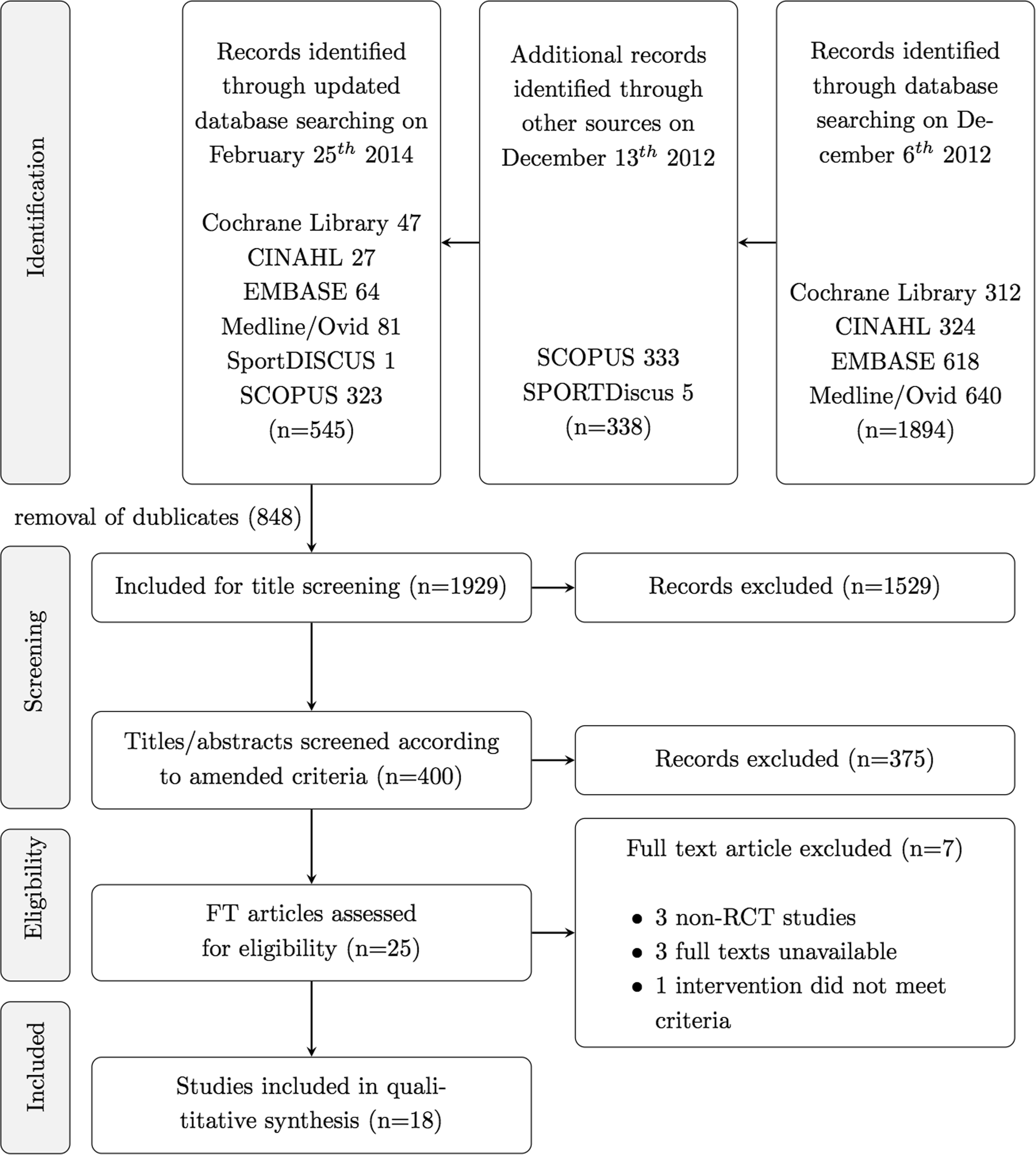

Supplement: Supplementary file 6 — Authors’ original file for figure 2 [file 12891_2014_2326_MOESM6_ESM.tif]
