# Supplementary material for: Effects of proprioceptive exercises on pain and function in chronic neck- and low back pain rehabilitation: a systematic literature review
Source: BMC Musculoskelet Disord. 2014 Nov 19;15:382. doi: 10.1186/1471-2474-15-382 (PMC4247630; doi:10.1186/1471-2474-15-382)
Supplement: Supplementary file 5 — Authors’ original file for figure 1 [file 12891_2014_2326_MOESM5_ESM.pdf]

	Random sequence generation (selection bias)	Allocation concealment (selection bias)	Blinding of participants and personnel (performance bias)	Blinding of outcome assessment (detection bias)	Incomplete outcome data (attrition bias)	Selective reporting (reporting bias)	Group similarity at baseline	Co–interventions	Compliance	Intention–to–treat–analysis	Timing of outcome assessments	Other bias
Beinert 2013	?	?	−	+	+	+	−	+	+	+	+	+
Chung 2013	?	?	−	?	+	+	+	+	+	−	+	+
Costa 2009	+	+	+	+	+	+	+	+	+	+	+	+
Frih 2009	?	?	−	?	?	+	+	−	+	+	+	+
Gatti 2011	?	−	−	+	+	+	−	+	+	+	+	+
Hudson 2010	+	+	−	+	+	+	−	−	+	+	+	+
Humphreys 2002	?	?	−	−	?	+	−	−	?	−	+	+
Jin 2013	+	?	−	−	+	+	+	+	+	+	+	+
Johannsen 1995	?	?	−	−	−	−	−	+	?	−	?	+
Jull 2007	?	?	−	+	−	+	+	+	?	−	+	+
Marshall 2008	?	?	−	?	−	+	+	+	?	−	+	+
Morone 2012	+	+	−	+	+	−	+	+	+	+	+	?
Paolucci 2012	?	?	−	?	?	+	?	+	+	−	+	+
Revel 1994	?	?	−	?	?	+	+	+	+	+	+	+
Sorensen 2010	?	+	−	+	+	+	+	−	+	+	+	+
Stankovic 2012	−	−	−	−	−	−	+	+	?	−	+	+
Suni 2006	?	?	−	+	+	−	?	−	+	+	+	+
Taimela 2000	?	?	−	+	+	−	+	−	?	+	+	+
